# Impact of Simulated Astigmatism on Visual Acuity, Stereopsis, and Reading in Young Adults

**DOI:** 10.3390/vision9040099

**Published:** 2025-12-16

**Authors:** Chow Wang Ming Shato, Ricardo Noguera Louzada, Pedro Lucas Machado Magalhães, Dillan Cunha Amaral, Daniel Oliveira Dantas, Daniel Alves Montenegro, Melanie May Chow, David Tayah, Milton Ruiz Alves

**Affiliations:** 1 Department of Ophthalmology and Otorhinolaryngology, Faculty of Medicine, University of São Paulo, São Paulo 05508-220, SP, Brazil; chowwang@usp.br (C.W.M.S.); damontenegro@usp.br (D.A.M.); milton.alves@hc.fm.usp.br (M.R.A.); 2 Department of Ophthalmology and Otorhinolaryngology, Faculty of Medicine, Federal University of Rio de Janeiro, Rio de Janeiro 21941-853, RJ, Brazil; dillanamaral@ufrj.br; 3 Faculty of Medicine, Institute of Medical Education, Angra dos Reis 23914-360, RJ, Brazil; pedrolucasm@mail.tau.ac.il; 4 Department of Computer Science, Federal University of Sergipe, São Cristóvão 49100-000, SE, Brazil; ddantas@dcomp.ufs.br; 5 School of Medicine, University of Santo Amaro, São Paulo 04829-300, SP, Brazil; melmayc12345@gmail.com; 6 Department of Clinical Surgery, Faculty of Medicine, Federal University of Amazonas, Manaus 69080-900, AM, Brazil; davidtayah@gmail.com

**Keywords:** astigmatism power, astigmatism axes, astigmatism visual acuity, binocular vision, astigmatism, reading

## Abstract

This study investigated the functional visual impact of simulated astigmatic blur using cylindrical powers of 0.50 D, 1.00 D, and 2.00 D, applied in against-the-rule (ATR), with-the-rule (WTR), and oblique (OBL) axes, in adults aged 18 to 35 years with no known ocular disease. Forty-five young adults were randomly divided into three groups (*n* = 15). Binocular best-corrected visual acuity (distance and near) was recorded in logMAR using the ETDRS acuity chart at 5 m and 40 cm, supported by acuity optotypes displayed in a Bailey–Lovie chart format. Depth-fusion and disparity discrimination were measured using polarized stereopsis thresholds with the Randot^®^ Stereo Test from Stereo Optical Company, Inc. Reading performance was quantified as a continuous binocular rate metric (words per minute) using the validated Portuguese digital reading curve provided by the MNREAD iPad App by Precision Vision at 40 cm. The results were preserved verbatim as follows: Distance and near BCVA were significantly affected by ATR astigmatisms (−0.50 + 1.00 90°, −1.00 + 2.00 90°), WTR astigmatisms (−0.25 + 0.50 180°, −0.50 + 1.00 180°, −1.00 + 2.00 180°), and OBL astigmatisms (OD: −0.25 + 0.50 45°, OS: −0.25 + 0.50 135°; OD: −0.50 + 1.00 45°, OS: −0.50 + 1.00 135°; OD: −1.00 + 2.00 45°, OS: −1.00 + 2.00 135°). Stereopsis was significantly influenced by high-power OBL astigmatism (−1.00 + 2.00). Reading rate was also negatively impacted by OBL astigmatisms ≥1.00 D. Simulated astigmatism of different powers and axes reduced high-contrast distance and near BCVA, stereopsis, and reading speed in adults aged 18–35 years. Higher-power astigmatism, particularly along oblique axes, caused the most significant functional impairment.

## 1. Introduction

Astigmatism is one of the most common refractive errors managed in clinical practice [1]. In an extensive systematic review and meta-analysis including 163 epidemiological studies worldwide, Hashemi et al. reported that astigmatism was highly prevalent across regions, affecting 14.9% of children and 40.4% of adults. In America, the area with the highest estimates among children, the pooled prevalence reached 27.2%, whereas Southeast Asia showed the lowest rates (9.8%) [1]. In adults, the highest prevalence of astigmatism was also found in the Americas (45.6%), followed closely by Southeast Asia (44.8%). This global synthesis highlights that astigmatism is consistently the most common refractive error across age groups, surpassing both myopia and hyperopia in overall frequency.

Uncorrected astigmatism influences both distance and near visual acuity (VA) [2,3,4,5,6]. With-the-rule astigmatism (WTR), also referred to as with-the-rule (WTR), describes cylindrical correction aligned near 180° in both eyes. VA is reduced to a greater extent when the axis of the crossed cylinder is either against-the-rule-astigmatism (ATR) and/or oblique (OBL) [7,8]. Knowledge of the influence of severity and subtypes of astigmatism on visual performance is essential for a better understanding of the impact of astigmatism on visual function [8].

It is well established that astigmatism leads to visual reduction at distance and near; however, the cutoff for clinically significant astigmatism has not been established. Results from Wang et al.’s large-scale population study showed that the prevalence of visual impairment increased substantially when the cylinder power was ≥1.00 diopter cylinder (DC), suggesting that this value may be an appropriate astigmatism cutoff [8]. Villegas et al. evaluated the minimum astigmatism required to achieve maximum visual performance and reported that astigmatism less than 0.50 DC did not degrade VA [9]. Previous studies, on the other hand, stressed the importance of correcting astigmatism, even for levels as low as 0.50 DC, since functional vision performance can be adversely affected even at these low levels of astigmatism [6,10].

The World Health Organization is aware of the barrier represented by the acquisition of eyeglasses. It encourages Member States to prioritize projects to distribute free or low-cost eyeglasses [11]. One way to expand effective refractive error coverage in the care of young adults would be, simultaneously with ophthalmological care, to include the dispensing of eyeglasses, which involves prescribing spherical lenses and correcting spherocylindrical errors in SE. Therefore, the first challenge that such conduct entails is to know the magnitude of the impact exerted on functional visual by different levels of power and orientation of the axis of the corrective cylinder of astigmatism that is no longer prescribed. The aim of this study was to investigate the effects of astigmatism power and axis on distance and near visual acuity, stereopsis, and reading rates in young adults.

## 2. Materials and Methods

Following approval of the study protocol by the Medical School of the University of São Paulo (Brazil) Ethics Committee, CAAE No. 29737320.8.0000.0068, informed consent was obtained from all participants.

The study population consisted of 45 young adults who presented spontaneously and were evaluated between January and September 2025. The inclusion criteria included: (1) no evidence of ocular disease; (2) both sexes; (3) aged between 18 and 35 years; (4) spherical refractive errors ≤ ±2.00 D without or with astigmatism ≤ −0.75 DC, and anisometropia with interocular difference in SE ≤ 0.50 diopters sphere (DS) (ocular refraction was performed under cycloplegia with the instillation of a drop of 1% tropicamide, Mydriacyl 1%, Novartis, Basel, Switzerland, and the examination was performed after 30 min); (5) best corrected visual acuity (BCVA) of 0.00 logMAR or better in both eyes; (6) normal binocular vision (orthophoria by cover/uncover test and presence of sensory fusion by the Bagolini striated lens test); (7) accommodation amplitude (AA) equal to or greater than that estimated by *Hoffstetter’s formula* (15 D−0.25 × age in years); (8) near point of convergence ≤ 10 cm; and (9) positive fusional vergence and negative fusional vergence with prism bar for distance and near, within normal values, distance and near, respectively, for convergence 45^Δ^/40^Δ^ and 25^Δ^/20^Δ^ and for divergence 14^Δ^/14^Δ^ and 8^Δ^/6^Δ^ [12].

Randomization was performed using a single-stage allocation procedure. All 45 participants were randomly assigned to one of the three study groups (ATR, WTR, or OBL astigmatism; *n* = 15 per group) using balanced block randomization with a block size of six to ensure equal group distribution. The random sequence was generated from a random-number table by a researcher not involved in data collection. Allocation concealment was maintained using sequentially numbered, sealed, opaque envelopes opened only after baseline measurements were completed. Both participants and the examiners responsible for visual acuity, stereopsis, and reading assessments were masked to group assignment during baseline testing.

All participants underwent cycloplegia during the baseline assessment. Afterward, each subject had their refractive error fully corrected in either spectacles or a trial frame, and baseline measurements of binocular distance BCVA, near BCVA, stereopsis, and reading rate were obtained. Before evaluating the effects of the three induced astigmatic conditions (ATR, WTR, and OBL; in three different powers) on these visual outcomes, participants underwent a 10 min adaptation period. During this period, they watched a movie on an iPad positioned at 40 cm while wearing the corresponding trial lenses, allowing sufficient adaptation to the astigmatic blur before formal testing. The duration of adaptation was based on the period required to adapt to astigmatic refractive blurring, as reported by Khan et al. (2013) [13].

We evaluated the effects of three astigmatic powers (0.50 DC, 1.00 DC, and 2.00 DC) applied in three orientations, ATR, WTR, and OBL, on distance and near visual acuity, stereopsis, and reading rate.

Group 1—ATR simulated astigmatism (*n* = 15). (1) baseline control condition; (2) −0.25 DS/+0.50 DC at 90° (both eyes); (3) −0.50 DS/+1.00 DC × 90° (both eyes); and (4) −1.00 DS/+2.00 DC × 90° (both eyes).

Group 2—WTR simulated astigmatism (*n* = 15). (1) baseline control condition; (2) −0.25 DS/+0.50 DC × 180° (both eyes); (3) −0.50 DS/+ 1.00 DC × 180° (both eyes); and (4) +1.00 DS/−2.00 DC × 90° (both eyes).

Group 3—OBL simulated astigmatism (*n* = 15). (1) baseline control condition; (2) OD: −0.25 DS/+0.50 DC × 45° and OS: −0.25 OD/+0.50 DC × 135°; (3) OD: −0.50 OD/+1.00 DC × 45° and OS: −0.50 OD/+1.00 DC × 135°; (4) OD: −1.00 OD/+2.00 DC × 45° and OS 1.00 OD/+2.00 DC × 135°.

The mean SE was maintained as plano for all simulated astigmatism conditions. Distance VA was measured with the ETDRS table at 5 m and near VA with the Bailey–Lovie table at 40 cm, in logMAR. The illumination and luminance of the EDRTS table for measuring distance vision were 650 lux and 192 cd/m^2^, respectively. The lighting and luminance of the Bailey–Lovie table for near-vision measurements were 689 lux and 196 cd/m^2^, respectively.

Stereoacuity was assessed using the Randot^®^ stereo test (Stereo Optical Company, Inc., Chicago, IL, USA). Participants wore polarized filters to isolate the images from each eye, thereby generating binocular disparity. The test was administered at the standard near distance of 40 cm, under the same dimly lit room conditions used in the reading protocol (ambient illumination of approximately 4.5 lux). Following the Randot procedure, the stopping rule was to identify the smallest disparity level correctly recognized, which was recorded as the stereoacuity threshold.

Reading rate (words per minute) was measured using the MNREAD test validated for the Portuguese language (MNREAD-P). Participants read binocularly at 40 cm, with sentences presented in decreasing print size steps of 0.1 log units. Photopic luminance was set to 220 cd/m^2^ (screen brightness 75), while mesopic luminance was 2 cd/m^2^, obtained with a neutral-density filter. The app automatically calibrated the angular print size and allowed the examiner to enter reading errors after each sentence. Standard MNREAD parameters were then automatically computed: maximum reading speed, critical print size, reading acuity, and reading accessibility index [14].

We included the criteria for repeating trials in cases of hesitation or loss of attention and described the procedure for excluding outliers based on intra-subject variability.

Ocular dominance was determined using the Miles test and recorded, although all reading measures were performed binocularly.

### Statistical Analysis

For the statistical analysis, the R Statistical Computing Environment (version 4.4.1; R Core Team, Vienna, Austria) was used [15].

Age was described using mean and standard deviation, and comparisons between groups were made using the Kruskal–Wallis test. The variable gender was described using absolute frequency, and the comparison between the groups was made using the Fisher test. The normality of the data distribution was verified using the Shapiro–Wilk test. A Friedman test, a nonparametric variant of the one-way repeated-measures ANOVA, was used because the assumption of normality did not hold. The Friedman test was used to evaluate the influence of the various dioptric powers of the simulated astigmatism on four variables: distance and near BCVA, stereopsis, and reading speed measured on the same subjects.

On the other hand, a Kruskal–Wallis test, which is a non-parametric variant of the one-way between-subjects ANOVA, was used, as each axis, ATR, WTR, and OBL, was measured on different subjects. The Kruskal–Wallis test was used to evaluate the influence of the three axes on the four variables measured on the different groups of subjects.

A *p*-value < 0.05 was considered statistically significant. Post hoc tests were performed when a considerable *p*-value was obtained.

A Wilcoxon paired test (i.e., Wilcoxon signed-rank test) was performed to assess the influence of dioptric power on the variables obtained from the same subjects. The Wilcoxon rank-sum test (unpaired Wilcoxon test) was used to assess the influence of the three astigmatic axes on outcomes across the different subject groups. To control for multiple comparisons, all resulting *p*-values were adjusted using the Holm–Bonferroni correction. Supplementary Tables S1–S12 show the adjusted *p*-values.

A previous study reported that the smallest difference in VA (logMAR) between a baseline group without lenses and a group with induced astigmatism of 1.0 DC was 0.8 [16]. The standard deviations of the VA in these groups were 0.06 and 0.09, respectively. Using these standard deviation values, a power analysis indicated that a sample size of 15 individuals would be enough for a two-sided test to detect a difference of 0.8 in the VA between the two groups with 0.8 power and a significance level of 0.05 (Supplementary Figure S1).

## 3. Results

Table 1 shows the distribution of participants across the three study groups by age and gender, and presents the static refraction parameters for the OD and OS in the three astigmatism groups, including spherical power, cylindrical power, and axis measurements.

**Figure 1:**
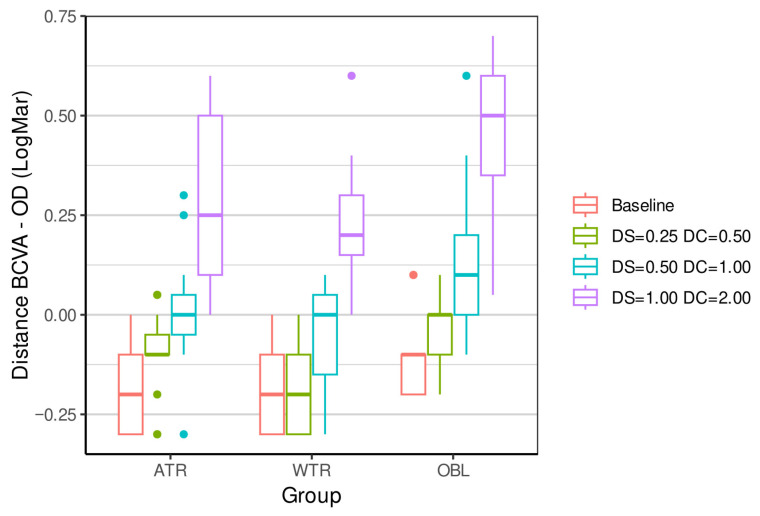
Boxplot shows the lower limit, 1st quartile, median, 3rd quartile, upper limit, and outliers of the measures for the three groups (ATR—against-the-rule astigmatism; WTR—with-the-rule astigmatism; OBL—oblique astigmatism) with different powers of simulated astigmatism on distance BCVA (logMAR) compared to the baseline. BCVA—best correct visual acuity; DS—diopters sphere; DC—diopters cylinder.

**Figure 2:**
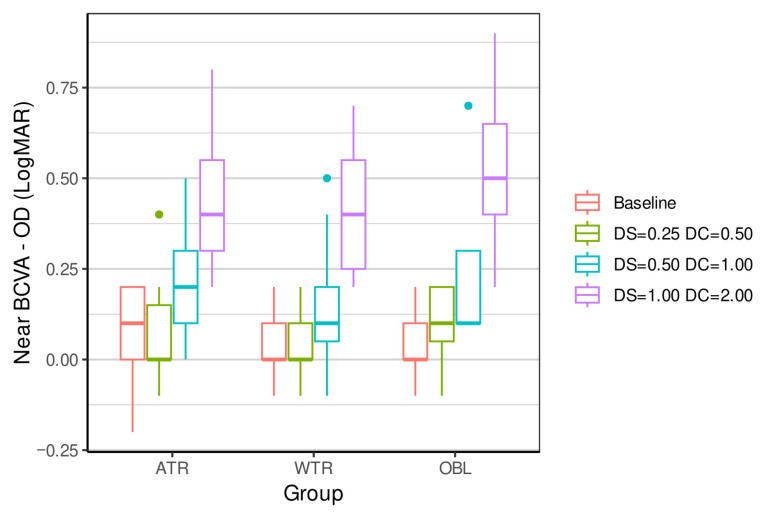
Same as Figure 1, but now showing results for near BCVA (logMAR) OD.

**Figure 3:**
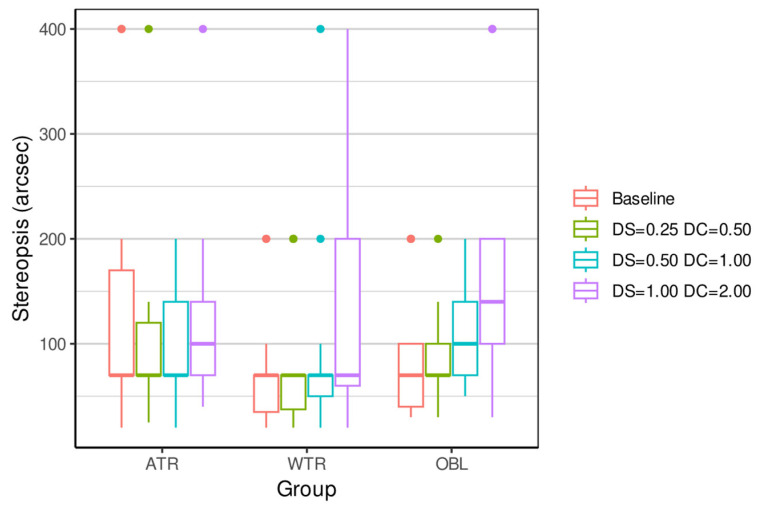
Same as Figure 1, but now showing results for stereopsis (Randot test) compared to baseline.

**Figure 4:**
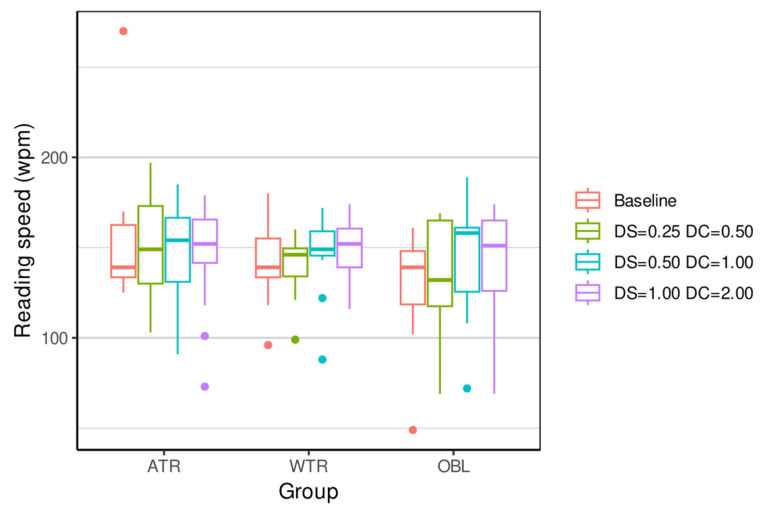
Same format as Figure 1, showing results for reading speed compared to baseline.

Table 2, Table 3, Table 4 and Table 5 and Figure 1, Figure 2, Figure 3 and Figure 4 show the result of the outcome measures of the distance and near BCVA (logMAR), stereopsis, and reading rate for the three levels of ATR, WTR, and OBL simulated astigmatisms compared to baseline.

Distance and near BCVA (baseline control) were significantly influenced by ATR simulated astigmatisms: −0.50 + 1.00 90° and −1.00 + 2.00 90°; WTR simulated astigmatisms: −0.25 + 0.50 180°, −0.50 + 1.00 180°, and −1.00 + 2.00 180°; and OBL simulated astigmatisms: OD: −0.25 + 0.50 45°, OS: −0.25 + 0.50 135°; OD: −0.50 + 1.00 45°, OS: −0.50 + 1.00 135°; and OD: −1.00 + 2.00 45°, OS: −1.00 + 2.00 135°.

Stereopsis (baseline control) was significantly influenced by OBL simulated astigmatism: OD: −1.00 + 2.00 45°, OS: −1.00 + 2.00 135° (*p* = 0.0337).

The reading rate (baseline control) was influenced by WTR simulated astigmatism −0.50 + 1.00 180° and by OBL simulated astigmatism OD: −0.50 + 1.00 45°, OS: −0.50 + 1.00 135° (*p* = 0.0011), and OD: −1.00 + 2.00 45°, OD: −1.00 + 2.00 135° (*p* = 0.0044).

## 4. Discussion

Astigmatic blur, particularly when induced along ATR and oblique axes, produced a consistent reduction in both distance and near BCVA in our sample. This pattern aligns with previous evidence showing that ATR astigmatism generally impairs visual acuity more than WTR blur, especially for tasks requiring higher spatial resolution and smaller optotypes [5,7,17,18]. Clinical studies similarly report that residual ATR astigmatism after cataract surgery leads to worse postoperative acuity compared with WTR residual error [18,19], and experimental work has shown that oblique astigmatism can degrade image quality even more severely than the horizontal and vertical meridians [19]. Taken together, these findings suggest that both the magnitude and orientation of astigmatic blur play an important role in determining the extent of acuity loss, with ATR and oblique axes producing the most significant functional impact.

A possible explanation for these axis-dependent differences is that horizontal retinal blur, typical of ATR and certain oblique astigmatisms, interferes more with stereopsis, since depth perception relies mainly on detecting horizontal disparities between the eyes’ images. Moreover, interocular asymmetry in astigmatic blur, especially with oblique axes, tends to disrupt binocular matching more severely than symmetric blur. By contrast, the minimal axis-related differences observed for visual acuity and reading performance may reflect that letter recognition depends on orientation components distributed across all meridians, making horizontal, vertical, and oblique blur similarly detrimental to optotype identification and reading speed.

In addition to axis orientation, astigmatism magnitude also influences visual loss, and several studies have explored the minimum cylinder power required to produce clinically relevant effects. Previous studies show that even low magnitudes of astigmatism, around 0.50 D, can negatively affect visual acuity, particularly in high-demand visual tasks and in patients undergoing cataract surgery with intraocular lenses. In line with this, studies indicate that correcting astigmatism of approximately 0.75 D or more leads to measurable improvements in visual performance, while residual cylinders as low as 0.50 D may still produce symptoms in sensitive or visually demanding situations [9,20,21,22,23]. However, the benefit of correcting very low, habitual natural astigmatism (<0.50 D) appears limited and highly subject-dependent, suggesting that naturally adapted cylinders in this range may not meaningfully affect day-to-day visual performance [24,25]. In healthy young individuals, the correction of astigmatism below 0.50 D typically yields minimal improvement [9]. In contrast, in older adults, uncorrected astigmatisms of 1.00 DC significantly decrease both distance and near visual acuity, impacting perceived clarity. Importantly, Sha et al. reported that prescribing spherical lenses in SE to correct astigmatism up to 0.50 DC has no significant effect on high-contrast VA, low-contrast VA, vision clarity, or vision satisfaction, although the tolerable axis misalignment increases as the cylinder magnitude decreases [7,26].

Stereopsis (*baseline*) was significantly influenced by simulated OBL astigmatism: −1.00 + 2.00 45°, −1.00 + 2.00 135° (*p* = 0.0337) (see Figure 3, Table 4 and Table S11). Uncorrected astigmatism affects depth perception because stereopsis depends on the clarity and symmetry of the images from both eyes [27,28]. Previous studies show that monocular or asymmetric blur impairs stereopsis more than equal binocular blur, highlighting the importance of interocular symmetry [27,28]. In our protocol, the oblique condition introduced opposite blur orientations between the eyes (45° vs. 135°), creating an interocular mismatch that likely amplified the stereoacuity loss observed [27]. Deepa et al. evaluated the stereo acuity levels using the random dot stereo acuity chart in 246 college students with 20 ± 1.9 years. They reported that 42.6% presented with reduced stereopsis (50–400 arcseconds) [29]. In other words, astigmatism, especially when bilateral, with high dioptric power or with orthogonal axes between the eyes, significantly compromises stereopsis. Correcting astigmatism adequately is essential to preserve binocular function and depth perception [30,31,32,33]. Clinically, these findings reinforce that unequal or monocular astigmatic blur has a disproportionately negative effect on stereopsis, and residual cylinder differences between eyes should be minimized to preserve depth perception and binocular comfort.

The reading rate (baseline) was influenced by simulated OBL astigmatisms OD: −0.50 + 1.00 45°, OS: −0.50 + 1.00 135° (*p* = 0.0010), and OD: −1.00 + 2.00 45°, OS: −1.00 + 2.00 135° (*p* = 0.0040) (see Figure 4, Table 5 and Table S12). In contrast, in a detailed study of the effects of astigmatism on reading rate, Wills et al. demonstrated that the refractive blurring of ATR and WTR, which simulates astigmatism, reduced the reading rate compared to the baseline [16]. Overall, most studies have shown that ATR astigmatism causes a greater loss of performance than WTR astigmatism or OBL [7,34,35].

### Limitations

One limitation of the study is the small number of participants per group (*n* = 15). Another limitation in extrapolating the effects of simulated astigmatism to real life is the sequenced performance of visual function tests: first, the measurement of distance and near BCVA, then stereopsis, and finally reading speed. In the study, after the interposition of lenses that simulate astigmatism and before each evaluation, to allow for the adaptation to astigmatic refractive blurring, each subject watched a video on the iPad placed 40 cm from the eyes for 10 min, with an interval of 5 min, and the clear vision after the conclusion of the test of each visual condition was evaluated. In real life, the human visual system adapts to astigmatic blurring, demonstrating functional plasticity that adjusts to both acute and chronic changes in the pattern of astigmatic blurring. Psychophysical studies have shown that, after brief or prolonged exposure to astigmatism, the oriented cortical filters are recalibrated, improving VA along the affected axis [36,37]. This adaptation is selective for the astigmatism axis, indicating that the visual system adjusts its response according to the predominant blurring pattern. In individuals with chronic astigmatism, evidence suggests the presence of automatic neural compensation, leading to more accurate orientation perception, even in the presence of uneven noise across meridians. This compensation persists even after full optical correction, suggesting that the visual system maintains long-lasting adaptations to habitual blurring [38,39].

## 5. Conclusions

The study evaluated the effects of simulated astigmatism with different powers (0.50, 1.00, and 2.00 D) and axes (90°, 180°, 45°, and 135°) on high-contrast distance and near BCVA, stereopsis, and reading speed in adults aged 18–35 years. Simulated astigmatism reduced not only high-contrast BCVA but also stereopsis and reading speed. Higher-power astigmatism, especially along oblique axes, produced the greatest negative impact on all visual functions tested.

## Figures and Tables

**Table 1:** Demographic data of the study participants and comparison of static refraction parameters (mean ± SD) among participants with ATR, WTR, and OBL astigmatism patterns.

	ATR ^1^			WTR ^2^			OBL ^3^			Kruskal *p*-Value
	Mean ± SD ^4^	Median	(Min, Max)	Mean ± SD ^4^	Median	(Min, Max)	Mean ± SD ^4^	Median	(Min, Max)	
Age	22.60 (4.82)	21	(18, 34)	25.27 (4.42)	25	(18, 32)	24.67 (5.92)	22	(18, 33)	0.2830
Static refraction, spherical, OD ^5^	0.16 (0.60)	0	(−0.75, 0.75)	0.21 (1.24)	0.25	(−2.00, 3.00)	0.06 (1.18)	0.25	(−2.00, 2.25)	0.9899
Static refraction, cylindrical, OD ^5^	−0.33 (0.18)	−0.25	(−0.75, 0.00)	−0.33 (0.12)	−0.25	(−0.50, 0.00)	−0.39 (0.24)	0	(−0.75, 0.00)	0.7955
Static refraction, axis, OD ^5^	−1.11 (37.40)	10	(−60.00, 45.00)	21.67 (52.74)	40	(−85.00, 90.00)	0.71 (75.80)	5	(−85.00, 85.00)	0.8957
Static refraction, spherical, OS ^6^	0.29 (0.45)	0.25	(−0.75, 1.00)	0.33 (1.01)	0.25	(−1.75, 2.75)	0.27 (0.92)	0.25	(−1.25, 2.50)	0.9732
Static refraction, cylindrical, OS ^6^	−0.40 (0.21)	−0.25	(−0.75, 0.00)	−0.50 (0.20)	0	(−0.75, 0.00)	−0.55 (0.21)	0	(−0.75, 0.00)	0.3439
Static refraction, axis, OS ^6^	4.50 (59.13)	0	(−85.00, 80.00)	33.57 (49.98)	55	(−50.00, 90.00)	−24.00 (65.13)	−35	(−85.00, 70.00)	0.2909
Gender	Fem./Male 9/6			Fem./Male 9/6			Fem./Male 5/10			Friedman *p*-value 0.1112

^1^ ATR—against-the-rule astigmatism; ^2^ WTR—with-the-rule astigmatism; ^3^ OBL—oblique astigmatism; ^4^ SD—standard deviation;^5^ OD—right eye; ^6^ OS—left eye.

**Table 2:** Results of the effects of simulated astigmatisms (group 1—ATR, group 2—WTR, and group 3—OBL) on distance best-corrected visual acuity (logMAR) compared to baseline control.

	Baseline			DS ^1^ = −0.25 DC ^2^ = 0.50			DS ^1^ = −0.50 DC ^2^ = 1.00			DS ^1^ = −1.00 DC ^2^ = 2.00			
	Mean ± SD ^3^	Median	(Min, Max)	Mean ± SD ^3^	Median	(Min, Max)	Mean ± SD ^3^	Median	(Min, Max)	Mean ± SD ^3^	Median	(Min, Max)	Friedman *p*-value
ATR ^4^	−0.18 (0.12)	−0.2	(−0.30, 0.00)	−0.10 (0.10)	−0.1	(−0.30, 0.05)	0.02 (0.14)	0	(−0.30, 0.30)	0.27 (0.21)	0.25	(0.00, 0.60)	<0.0001 ***
WTR ^5^	−0.21 (0.10)	−0.2	(−0.30, 0.00)	−0.18 (0.10)	−0.2	(−0.30, 0.00)	−0.05 (0.13)	0	(−0.30, 0.10)	0.22 (0.15)	0.2	(0.00, 0.60)	<0.0001 ***
OBL ^6^	−0.11 (0.10)	−0.1	(−0.20, 0.10)	−0.03 (0.08)	0	(−0.20, 0.10)	0.14 (0.18)	0.1	(−0.10, 0.60)	0.44 (0.18)	0.5	(0.05, 0.70)	<0.0001 ***
Kruskal *p*-value	0.0715			0.0026 **			0.0128 *			0.0096 **			

*** < 0.001; ** < 0.01; * < 0.05. ^1^ DS—diopters sphere; ^2^ DC—diopters cylinder; ^3^ SD—standard deviation; ^4^ ATR—against-the-rule astigmatism; ^5^ WTR—with-the-rule astigmatism; ^6^ OBL—oblique astigmatism.

**Table 3:** Results of the effects of simulated astigmatisms (group 1—ATR, group 2—WTR, and group 3—OBL) on near best-corrected visual acuity (logMAR) compared to baseline control.

	Baseline			DS ^1^ = −0.25 DC ^2^ = 0.50			DS ^1^ = −0.50 DC ^2^ = 1.00			DS ^1^ = −1.00 DC ^2^ = 2.00			
	Mean ± SD ^3^	Median	(Min, Max)	Mean ± SD ^3^	Median	(Min, Max)	Mean ± SD ^3^	Median	(Min, Max)	Mean ± SD ^3^	Median	(Min, Max)	Friedman *p*-value
ATR ^4^	0.07 (0.12)	0.1	(−0.20, 0.20)	0.08 (0.13)	0	(−0.10, 0.40)	0.21 (0.15)	0.2	(0.00, 0.50)	0.43 (0.16)	0.4	(0.20, 0.80)	<0.0001 ***
WTR ^5^	0.04 (0.10)	0	(−0.10, 0.20)	0.03 (0.09)	0	(−0.10, 0.20)	0.14 (0.16)	0.1	(−0.10, 0.50)	0.41 (0.17)	0.4	(0.20, 0.70)	<0.0001 ***
OBL ^6^	0.03 (0.08)	0	(−0.10, 0.20)	0.11 (0.10)	0.1	(−0.10, 0.20)	0.20 (0.16)	0.1	(0.10, 0.70)	0.51 (0.20)	0.5	(0.20, 0.90)	<0.0001 ***
Kruskal *p*-value	0.5749			0.1346			0.264			0.3633			

*** < 0.001; ^1^ DS—diopters sphere; ^2^ DC—diopters cylinder; ^3^ SD—standard deviation; ^4^ ATR—against-the-rule astigmatism; ^5^ WTR—with-the-rule astigmatism; ^6^ OBL—oblique astigmatism.

**Table 4:** Results of the effects of simulated astigmatisms (group 1—ATR, group 2—WTR, and group 3—OBL) on stereopsis (arc seconds) compared to baseline control.

	Baseline			DS ^1^ = −0.25 DC ^2^ = 0.50			DS ^1^ = −0.50 DC ^2^ = 1.00			DS^1^ = −1.00 DC^2^ = 2.00			
	Mean ± SD ^3^	Median	(Min, Max)	Mean ± SD ^3^	Median	(Min, Max)	Mean ± SD ^3^	Median	(Min, Max)	Mean ± SD ^3^	Median	(Min, Max)	Friedman *p*-value
ATR ^4^	134.67 (118.68)	70	(20, 400)	104.33 (89.90)	70	(25, 400)	100.67 (56.37)	70	(20, 200)	125.33 (91.02)	100	(40, 400)	0.2419
WTR ^5^	73.33 (56.65)	70	(20, 200)	79.00 (65.58)	70	(20, 200)	89.00 (96.18)	70	(20, 400)	123.33 (99.76)	70	(20, 400)	0.0047 **
OBL ^6^	86.67 (62.30)	70	(30, 200)	84.67 (45.80)	70	(30, 200)	110.00 (53.18)	100	(50, 200)	166.00 (108.94)	140	(30, 400)	0.0002 ***
Kruskal *p*-value	0.1206			0.2953			0.1072			0.2694			

*** <0.001; ** <0.01; ^1^ DS—diopters sphere; ^2^ DC—diopters cylinder; ^3^ SD—standard deviation; ^4^ ATR—against-the-rule astigmatism; ^5^ WTR—with-the-rule astigmatism; ^6^ OBL—oblique astigmatism.

**Table 5:** Results of the effects of simulated astigmatisms (group 1—ATR, group 2—WTR, and group 3—OBL) on reading rate compared to baseline control.

	Baseline			DS ^1^ = −0.25 DC ^2^ = 0.50			DS ^1^ = −0.50 DC ^2^ = 1.00			DS ^1^ = −1.00 DC ^2^ = 2.00			
	Mean ± SD ^3^	Median	(Min, Max)	Mean ± SD ^3^	Median	(Min, Max)	Mean ± SD ^3^	Median	(Min, Max)	Mean ± SD ^3^	Median	(Min, Max)	Friedman *p*-value
ATR ^4^	153.27 (35.79)	139	(125, 270)	149.07 (25.72)	149	(103, 197)	147.40 (26.94)	154	(91, 185)	146.73 (29.59)	152	(73, 179)	0.3761
WTR ^5^	142.40 (21.52)	139	(96, 180)	141.53 (15.96)	146	(99, 160)	147.53 (20.30)	149	(88, 172)	148.27 (17.80)	152	(116, 174)	0.5641
OBL ^6^	128.67 (28.32)	139	(49, 161)	136.53 (29.40)	132	(69, 169)	144.53 (29.83)	158	(72, 189)	142.73 (28.57)	151	(69, 174)	<0.0001 ***
Kruskal *p*-value	0.274			0.4963			0.9819			0.9079			

*** < 0.001; ^1^ DS—diopters sphere; ^2^ DC—diopters cylinder; ^3^ SD—standard deviation; ^4^ ATR—against-the-rule astigmatism; ^5^ WTR—with-the-rule astigmatism; ^6^ OBL—oblique astigmatism.

## Data Availability

The original contributions presented in this study are included in the article/Supplementary Materials. Further inquiries can be directed to the corresponding author.

## Supplementary Materials

The following supporting information can be downloaded at: https://www.mdpi.com/article/10.3390/vision9040099/s1, Figure S1: A power analysis indicated that a sample size of 15 individuals would be enough for a two-sided test to detect a difference of 0.8 in the VA between the two groups with 0.8 power and a significance level of 0.05; Table S1: Post-hoc tests of Distance BCVA-OD (LogMar) in group ATR. The *p*-values were adjusted by the Holm-Bonferroni method; Table S2: Post-hoc tests of Distance BCVA-OD (LogMar) in group OBL. The *p*-values were adjusted by the Holm-Bonferroni method; Table S3: Post-hoc tests of Distance BCVA-OD (LogMar) in group WTR. The *p*-values were adjusted by the Holm-Bonferroni method; Table S4: Post-hoc tests of Distance BCVA-OD (LogMar) with power DS = −0.25 DC = 0.50. The *p*-values were adjusted by the Holm-Bonferroni method; Table S5: Post-hoc tests of Distance BCVA-OD (LogMar) with power DS = −0.50 DC = 1.00. The *p*-values were adjusted bythe Holm-Bonferroni method; Table S6: Post-hoc tests of Distance BCVA-OD (LogMar) with power DS = −1.00 DC = 2.00. The *p*-values were adjusted by the Holm-Bonferroni method; Table S7: Post-hoc tests of Near BCVA-OD (LogMar) in group ATR. The *p*-values were adjusted by the Holm-Bonferroni method; Table S8: Post-hoc tests of Near BCVA-OD (LogMar) in group OBL. The *p*-values were adjusted by the Holm-Bonferroni method; Table S9: Post-hoc tests of Near BCVA-OD (LogMar) in group WTR. The *p*-values were adjusted by the Holm-Bonferroni method; Table S10: Post-hoc tests of Stereopsis (arcsec) in group WTR. The *p*-values were adjusted by the Holm-Bonferroni method; Table S11: Post-hoc tests of Stereopsis (arcsec) in group OBL. The *p*-values were adjusted by the Holm-Bonferroni method; Table S12: Post-hoc tests of Reading speed (wpm) in group OBL. The *p*-values were adjusted by the Holm-Bonferroni method.

## Abbreviations

The following abbreviations are used in this manuscript:

VA	Visual acuity
ATR	Against-the-rule astigmatism
OBL	Oblique astigmatism
WTR	With-the-rule astigmatism
DC	Diopters of cylinder
SE	Spherical equivalent
DS	Spherical diopter
OD	Oculus dexter (right eye)
OS	Oculus sinister (left eye)
BCVA	Best-corrected visual acuity
AA	Amplitude of accommodation
logMAR	Logarithm of the minimum angle of resolution
ETDRS	Early treatment diabetic retinopathy study
MNREAD-P	Minnesota Reading Test, Portuguese version
ANOVA	Analysis of variance
SD	Standard deviation
