# Supplementary material for: Impact of Simulated Astigmatism on Visual Acuity, Stereopsis, and Reading in Young Adults
Source: Vision (Basel). 2025 Dec 16;9(4):99. doi: 10.3390/vision9040099 (PMC12737380; doi:10.3390/vision9040099)
Supplement: Supplementary file 1 [file vision-09-00099-s001.zip › vision-3941508-supplementary.pdf]

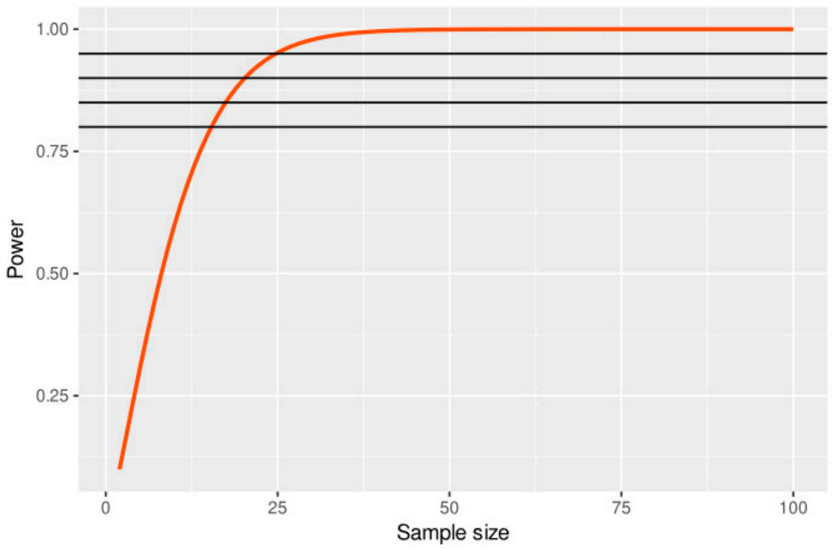

Figure S1. A power analysis indicated that a sample size of 15 individuals would be enough for a two-sided test to detect a difference of 0.8 in the VA between the two groups with 0.8 power and a significance level of 0.05.

Distance BCVA - OD (LogMAR)

Table S1. Post-hoc tests of Distance BCVA - OD (LogMar) in group ATR. The p-values were adjusted by the Holm-Bonferroni method.

Group 1	Group 2	Effect	95% CI	p-value	p adj.
Baseline	DS=-0.25 DC=0.50	0.49	(0.06 - 0.78)	0.0370*	0.0370*
Baseline	DS=-0.50 DC=1.00	0.75	(0.42 - 0.88)	0.0040**	0.0100*
Baseline	DS=-1.00 DC=2.00	0.87	(0.84 - 0.89)	0.0010**	0.0050**
DS=-0.25 DC=0.50	DS=-0.50 DC=1.00	0.82	(0.68 - 0.89)	0.0030**	0.0100*
DS=-0.25 DC=0.50	DS=-1.00 DC=2.00	0.88	(0.88 - 0.89)	0.0007***	0.0040**
DS=-0.50 DC=1.00	DS=-1.00 DC=2.00	0.88	(0.84 - 0.89)	0.0010**	0.0050**

*** <0.001; ** <0.01; * <0.05.

Table S2. Post-hoc tests of Distance BCVA - OD (LogMar) in group OBL. The p-values were adjusted by the Holm-Bonferroni method.

Group 1	Group 2	Effect	95% CI	p-value	p adj.
Baseline	DS=-0.25 DC=0.50	0.24	(0.01 - 0.63)	0.2920	0.2920
Baseline	DS=-0.50 DC=1.00	0.77	(0.52 - 0.88)	0.0050**	0.0140*
Baseline	DS=-1.00 DC=2.00	0.88	(0.84 - 0.9)	0.0010**	0.0050**
DS=-0.25 DC=0.50	DS=-0.50 DC=1.00	0.69	(0.34 - 0.86)	0.0130*	0.0250*
DS=-0.25 DC=0.50	DS=-1.00 DC=2.00	0.88	(0.88 - 0.89)	0.0007***	0.0040**
DS=-0.50 DC=1.00	DS=-1.00 DC=2.00	0.88	(0.84 - 0.89)	0.0010**	0.0050**

*** <0.001; ** <0.01; * <0.05.

Table S3. Post-hoc tests of Distance BCVA - OD (LogMar) in group WTR. The p-values were adjusted by the Holm-Bonferroni method.

Group 1	Group 2	Effect	95% CI	p-value	p adj.
Baseline	DS=-0.25 DC=0.50	0.70	(0.38 - 0.89)	0.0090**	0.0090**
Baseline	DS=-0.50 DC=1.00	0.88	(0.88 - 0.9)	0.0007***	0.0040**
Baseline	DS=-1.00 DC=2.00	0.88	(0.88 - 0.89)	0.0007***	0.0040**
DS=-0.25 DC=0.50	DS=-0.50 DC=1.00	0.81	(0.62 - 0.9)	0.0020**	0.0040**
DS=-0.25 DC=0.50	DS=-1.00 DC=2.00	0.88	(0.88 - 0.9)	0.0007***	0.0040**
DS=-0.50 DC=1.00	DS=-1.00 DC=2.00	0.88	(0.84 - 0.89)	0.0010**	0.0040**

*** <0.001; ** <0.01; * <0.05.

Table S4. Post-hoc tests of Distance BCVA - OD (LogMar) with power DS=-0.25 DC=0.50. The p-values were adjusted by the Holm-Bonferroni method.

Group 1	Group 2	Effect	95% CI	p-value	p adj.
ATR	WTR	0.58	(0.16 - 0.87)	0.0460*	0.0920
ATR	OBL	0.38	(0.03 - 0.75)	0.1120	0.1120
WTR	OBL	0.70	(0.37 - 0.88)	0.0009***	0.0030**

*** <0.001; ** <0.01; * <0.05.

Table S5. Post-hoc tests of Distance BCVA - OD (LogMar) with power DS=-0.50 DC=1.00. The p-values were adjusted by the Holm-Bonferroni method.

Group 1	Group 2	Effect	95% CI	p-value	p adj.
ATR	WTR	0.33	(0.02 - 0.73)	0.2830	0.2830
ATR	OBL	0.46	(0.06 - 0.82)	0.0620	0.1230
WTR	OBL	0.75	(0.47 - 0.88)	0.0040**	0.0130*

*** <0.001; ** <0.01; * <0.05.

Table S6. Post-hoc tests of Distance BCVA - OD (LogMar) with power DS=-1.00 DC=2.00. The p-values were adjusted by the Holm-Bonferroni method.

Group 1	Group 2	Effect	95% CI	p-value	p adj.
ATR	WTR	0.04	(0.01 - 0.58)	0.5860	0.5860
ATR	OBL	0.54	(0.14 - 0.84)	0.0320*	0.0640
WTR	OBL	0.63	(0.21 - 0.88)	0.0030**	0.0090**

*** <0.001; ** <0.01; * <0.05.

Near BCVA - OD (LogMAR)

Table S7. Post-hoc tests of Near BCVA - OD (LogMar) in group ATR. The p-values were adjusted by the Holm-Bonferroni method.

Group 1	Group 2	Effect	95% CI	p-value	p adj.
Baseline	DS=-0.25 DC=0.50	0.09	(0.01 - 0.58)	0.7640	0.7640
Baseline	DS=-0.50 DC=1.00	0.68	(0.34 - 0.86)	0.0140*	0.0280*
Baseline	DS=-1.00 DC=2.00	0.88	(0.84 - 0.89)	0.0010**	0.0040**
DS=-0.25 DC=0.50	DS=-0.50 DC=1.00	0.82	(0.72 - 0.88)	0.0040**	0.0110*
DS=-0.25 DC=0.50	DS=-1.00 DC=2.00	0.88	(0.88 - 0.89)	0.0007***	0.0040**
DS=-0.50 DC=1.00	DS=-1.00 DC=2.00	0.88	(0.88 - 0.89)	0.0007***	0.0040**

*** <0.001; ** <0.01; * <0.05.

Table S8. Post-hoc tests of Near BCVA - OD (LogMar) in group OBL. The p-values were adjusted by the Holm-Bonferroni method.

Group 1	Group 2	Effect	95% CI	p-value	p adj.
Baseline	DS=-0.25 DC=0.50	0.03	(0 - 0.56)	0.8210	0.8210
Baseline	DS=-0.50 DC=1.00	0.61	(0.21 - 0.85)	0.0400*	0.0790
Baseline	DS=-1.00 DC=2.00	0.88	(0.88 - 0.9)	0.0007***	0.0040**
DS=-0.25 DC=0.50	DS=-0.50 DC=1.00	0.72	(0.51 - 0.84)	0.0140*	0.0410*
DS=-0.25 DC=0.50	DS=-1.00 DC=2.00	0.88	(0.88 - 0.9)	0.0007***	0.0040**
DS=-0.50 DC=1.00	DS=-1.00 DC=2.00	0.88	(0.88 - 0.89)	0.0007***	0.0040**

*** <0.001; ** <0.01; * <0.05.

Table S9. Post-hoc tests of Near BCVA - OD (LogMar) in group WTR. The p-values were adjusted by the Holm-Bonferroni method.

Group 1	Group 2	Effect	95% CI	p-value	p adj.
Baseline	DS=-0.25 DC=0.50	0.66	(0.29 - 0.88)	0.0150*	0.0300*
Baseline	DS=-0.50 DC=1.00	0.88	(0.84 - 0.92)	0.0009***	0.0040**
Baseline	DS=-1.00 DC=2.00	0.88	(0.88 - 0.9)	0.0007***	0.0040**
DS=-0.25 DC=0.50	DS=-0.50 DC=1.00	0.55	(0.19 - 0.84)	0.0430*	0.0430*
DS=-0.25 DC=0.50	DS=-1.00 DC=2.00	0.88	(0.88 - 0.9)	0.0007***	0.0040**
DS=-0.50 DC=1.00	DS=-1.00 DC=2.00	0.87	(0.84 - 0.89)	0.0010**	0.0040**

*** <0.001; ** <0.01; * <0.05.

Stereopsis (arcsec)

Table S10. Post-hoc tests of Stereopsis (arcsec) in group WTR. The p-values were adjusted by the Holm-Bonferroni method.

Group 1	Group 2	Effect	95% CI	p-value	p adj.
Baseline	DS=-0.25 DC=0.50	0.01	(0 - 0.41)	0.9660	1.0000
Baseline	DS=-0.50 DC=1.00	0.04	(0 - 0.42)	0.8300	1.0000
Baseline	DS=-1.00 DC=2.00	0.29	(0.02 - 0.61)	0.1150	0.6900
DS=-0.25 DC=0.50	DS=-0.50 DC=1.00	0.06	(0 - 0.42)	0.7480	1.0000
DS=-0.25 DC=0.50	DS=-1.00 DC=2.00	0.28	(0.02 - 0.58)	0.1260	0.6900
DS=-0.50 DC=1.00	DS=-1.00 DC=2.00	0.24	(0.02 - 0.57)	0.1870	0.7480

*** <0.001; ** <0.01; * <0.05.

Table S11. Post-hoc tests of Stereopsis (arcsec) in group OBL. The p-values were adjusted by the Holm-Bonferroni method.

Group 1	Group 2	Effect	95% CI	p-value	p adj.
Baseline	DS=-0.25 DC=0.50	0.13	(0 - 0.63)	0.8880	0.8880
Baseline	DS=-0.50 DC=1.00	0.63	(0.24 - 0.87)	0.0320*	0.0950
Baseline	DS=-1.00 DC=2.00	0.75	(0.46 - 0.88)	0.0060**	0.0340*
DS=-0.25 DC=0.50	DS=-0.50 DC=1.00	0.67	(0.45 - 0.82)	0.0220*	0.0890
DS=-0.25 DC=0.50	DS=-1.00 DC=2.00	0.73	(0.44 - 0.88)	0.0080**	0.0380*
DS=-0.50 DC=1.00	DS=-1.00 DC=2.00	0.55	(0.13 - 0.82)	0.0320*	0.0950

*** <0.001; ** <0.01; * <0.05.

Reading speed (wpm)

Table S12. Post-hoc tests of Reading speed (wpm) in group OBL. The p-values were adjusted by the Holm-Bonferroni method.

Group 1	Group 2	Effect	95% CI	p-value	p adj.
Baseline	DS=-0.25 DC=0.50	0.57	(0.16 - 0.88)	0.0310*	0.1000
Baseline	DS=-0.50 DC=1.00	0.85	(0.73 - 0.88)	0.0002***	0.0010**
Baseline	DS=-1.00 DC=2.00	0.87	(0.79 - 0.88)	0.0009***	0.0040**
DS=-0.25 DC=0.50	DS=-0.50 DC=1.00	0.42	(0.04 - 0.78)	0.1110	0.2220
DS=-0.25 DC=0.50	DS=-1.00 DC=2.00	0.61	(0.18 - 0.88)	0.0250*	0.1000
DS=-0.50 DC=1.00	DS=-1.00 DC=2.00	0.08	(0.01 - 0.57)	0.6750	0.6750

*** <0.001; ** <0.01; * <0.05.
